# The Triglyceride-Glucose Index as a Marker in Idiopathic Restless Legs Syndrome

**DOI:** 10.7759/cureus.71255

**Published:** 2024-10-11

**Authors:** Gülhan Sarıçam, Fahrettin Ege, Memet Aslanyavrusu

**Affiliations:** 1 Neurology, Pursaklar State Hospital, Ankara, TUR; 2 Neurology, Yüksek İhtisas University, Ankara, TUR; 3 Neurology, Kayseri Training and Research Hospital, Kayseri, TUR

**Keywords:** body mass index, diabetes mellitus, obesity, restless legs syndrome, triglyceride-glucose index

## Abstract

Background and objective

Restless legs syndrome (RLS) is more common in diabetic patients than in the general population. In this study, we aimed to explore the association between idiopathic RLS and the triglyceride-glucose (TyG) index, which is a simple and accessible marker of insulin resistance (IR).

Materials and methods

The study included 200 patients diagnosed with RLS according to the International Restless Study Group criteria, as well as 112 healthy volunteers. We interpreted the variables related to TyG and RLS by using the Pearson correlation coefficient and independent samples t-test. Multivariate linear regression analyses and receiver operating characteristic (ROC) curve analyses were employed to demonstrate the discriminatory power of the variables.

Results

The mean age of the patients with RLS was 44.73 ±11.29 years, and 71.5% (n=43) were female. The level of triglycerides, as well as BMI and TyG index, were significantly higher in patients with RLS compared to the control group (p=0.040, p=0.001, p=0.019, respectively). The TyG index showed a positive correlation with both disease duration (months) and BMI (r=0.281, p=0.000; r=0.507, p=0.000). The results of the multivariate regression analysis indicated a linear relationship between the TyG index and increasing RLS severity and BMI (β=0.286, t=4.199, p=0.000; β=0.041, t=6.754, p=0.000).

Conclusions

Our study revealed a direct correlation between the TyG index and increased disease severity, disease duration, and BMI in RLS. The findings indicate that the TyG index may serve as a valuable marker for RLS severity and high BMI.

## Introduction

Restless legs syndrome (RLS) is a chronic neurosensorimotor disorder characterized by a sense of relief during movement or walking, often accompanied by uncomfortable or unpleasant sensations, and a worsening of symptom severity at night [1]. Even though the pathophysiology of this condition is not fully understood, studies have demonstrated the significant roles played by the dysfunction of dopamine cells in the nigrostriatal regions, iron deficiency in the brain, and genetic components in the pathogenesis of RLS [2]. Additionally, research has shown that RLS is more common in people with kidney disease, rheumatoid arthritis, iron deficiency, and pregnancy. It has also been linked to migraines, some cardiovascular diseases, and diabetes [3].

Diabetes mellitus is a multifactorial metabolic disease caused by the body's inability to produce insulin or insulin resistance (IR), and it affects approximately 425 million people worldwide [4]. Several studies have indicated that factors contributing to diabetes, such as age, lifestyle, exercise, and BMI, are also associated with RLS [5]. A meta-analysis by Ning et al. involving 31 studies concluded that the prevalence of RLS in individuals with diabetes was more than two-fold when compared to the general population [6]. Furthermore, Bener et al. have observed significantly elevated HbA1c levels in patients with RLS compared to those without RLS in a cross-sectional study of patients with type 2 diabetes mellitus (T2DM) [7]. Studies on the relationship between diabetes and RLS have led us to hypothesize that patients with idiopathic RLS may also have glucose metabolism disorder.

Pre-diabetes mellitus refers to the intermediate phase between normal blood sugar levels and the onset of diabetes, characterized by impaired glucose metabolism and often IR [8]. In addition to the limitations of the homeostasis model in IR assessment, the gold standard euglycemic clamp test is time-consuming and challenging to perform [9]. Recently, the triglyceride-glucose (TyG) index, calculated from fasting triglyceride (TG) and fasting plasma glucose (FPG) levels, has been demonstrated to be a simple and reliable marker of IR [10]. In light of this, we aimed to investigate the relationship between the TyG index, demographic characteristics, and disease severity in patients with idiopathic RLS to gain a deeper understanding of the links between RLS and diabetes.

## Materials and methods

Study design and participants

This retrospective study was approved by the Yüksek İhtisas University Ethics Committee (E-2024/05/12). A total of 200 patients diagnosed with RLS and admitted to the neurology outpatient clinic between January 2020 and March 2024 were included in the study. We excluded patients with chronic diseases (diabetes, hypertension, thyroid, heart, lung, chronic liver, kidney, and hematologic diseases), acute or chronic inflammatory disease, oncologic disease, immunodeficiency, pregnancy, drug use, and infections in the last 30 days. Patients with deficiencies in biochemical data or anamnesis were also excluded. The monotherapy group included patients who were administered one of the preparations as a form of treatment, including pregabalin, dopamine agonists, iron, and vitamins. Conversely, those who were administered multiple drugs were included in the polytherapy group. The control group consisted of 112 healthy volunteers who presented themselves at the outpatient clinic for a routine check-up and were free of any disease or drug use.

We recorded the demographic characteristics, fasting blood glucose (FBG), and blood lipid parameters of the patient and control groups. The TyG index and BMI of the individuals were calculated using the formula [fasting TG (mg/dl) x FBG (mg/dl)/2] and weight (kg)/height (m^2^) [11].

Patients who were diagnosed with RLS according to the RLS diagnostic criteria and applied the RLS Severity Rating Scale [determined by the International RLS Study Group (IRLSSG)] were included in the study [12]. The RLS severity rating scale score ranges from 0 to 40 and consists of 10 questions. Grade 1: mild disease, 1 to 10; grade 2: moderate disease 11 to 20; grade 3: severe disease, 21 to 30; grade 4: very severe disease, 31-40 [13]. Patients whose data could not be obtained were excluded from the study.

Statistical analysis

The study population consisted of 312 individuals, including 200 patients and 112 healthy controls. SPSS Statistics 26 package software (IBM Corp., Armonk, NY) was used to analyze the data. While evaluating the study data, descriptive statistics [mean, standard deviation (SD)] were used for describing numerical variables, while frequencies (percentage, number) were employed for categorical variables. It was determined that the variables were normally distributed, as the coefficients fell within the range of ±2, and parametric statistical methods were employed.

The relationships between two independent numerical variables were analyzed using the Pearson correlation coefficient. Differences between two independent groups, such as biochemical changes between the RLS and control groups, were evaluated by the independent samples t-test. We evaluated differences between more than two independent groups, such as RLS severity and TyG index, using one-way analysis of variance (ANOVA). In the event of a discrepancy resulting from ANOVA, we employed the Tukey test to determine its origin. The multiple linear regression model was utilized to investigate the effect of more than one independent variable on a dependent numerical variable, while the receiver operating characteristic (ROC) curve analysis was employed to determine whether a numerical variable is a discriminating variable in diagnosis. A p-value <0.05 was considered statistically significant.

## Results

The mean age of patients with RLS was 44.73 ±11.29 years, and 71.5% (n=143) were female. The mean disease duration was 14.03 ±10.22 months, with 82% (n=164) of patients receiving monotherapy. The RLS was mildly severe (grade 1) in 33.5% (n=67), moderate (grade 2) in 29% (n=58), severe (grade 3) in 21.5% (n=43), and very severe (grade 4) in 16% (n=32). There was no statistically significant difference between the groups in terms of gender as per the chi-square analysis and age based on the independent samples t-test (p>0.05) (Table 1).

**Table 1:** Demographic characteristics of the patients by groups t: independent samples t-test RLS: restless legs syndrome; SD: standard deviation

Variable	RLS (n=200)	Control (n=112)	Chi-square	p	
	N (%)	N (%)			
Gender			0.702	0.402	
Female	143 (71.5)	75 (67)			
Male	57 (28.5)	37 (33)			
Medication					
Monotherapy	164 (82)	NA			
Polytherapy	36 (18)	NA			
RLS severity					
Grade 1	67 (33.5)	NA			
Grade 2	58 (29.0)	NA			
Grade 3	43 (21.5)	NA			
Grade 4	32 (16.0)	NA			
	Mean ±SD	Mean ±SD	t	p	
Age (years)	44.73 ±11.29	42.06 ±12.25	1.897	0.059	
Disease duration (months)	14.03 ±10.22	NA			

We analyzed biochemical changes between the control and RLS groups using an independent sample t-test. TyG index, BMI, and triglyceride levels were significantly higher in patients with RLS (p=0.019, p=0.001, and p=0.040 respectively). No significant differences were observed between the groups with regard to FBG, HDL, and LDL values (p<0.05) (Table 2).

**Table 2:** Biochemical test results and differences by groups *P<0.05; t: independent samples t-test BMI: body mass index; FBG: fasting blood glucose; HDL: high-density lipoprotein cholesterol; LDL: low-density lipoprotein cholesterol; RLS: restless legs syndrome; SD: standard deviation; TG: triglycerides; TyG: triglyceride-glucose index

Variable	RLS (n=200)	Control (n=112)	t	p
	Mean ±SD	Mean ±SD		
FBG (mg/dl)	94.13 ±8.47	92.86 ±8.15	1.285	0.200
TG (mg/dI)	139.93 ±64.54	124.55 ±61.05	2.058	0.040*
HDL (mg/dI)	51.11 ±15.11	53.32 ±11.71	-1.441	0.151
LDL (mg/dI)	107.23 ±33.07	101.973 ±27.59	1.425	0.155
BMI	25.34 ±4.72	23.70 ±3.83	3.325	0.001*
TyG	8.69 ±0.47	8.55 ±0.47	2.362	0.019*

The statistical analysis revealed no significant correlation between gender, medication, and TyG index in patients with RLS (p>0.05). A low positive correlation was found between the TyG index and disease duration (months; r=0.281, p=0.000). On the other hand, we observed a moderate correlation (r=0.507, p=0.000) between the TyG index and BMI. We employed a one-way ANOVA to evaluate the relationship between disease severity and TyG index in patients with RLS. The TyG index was found to be higher in patients with RLS severity levels 3 and 4 compared to patients with RLS severity levels 1 and 2 (F=15.871, p=0.000) (Table 3).

**Table 3:** The association between TyG index and variables in RLS *P<0.05; Difference: Tukey; F: one-way analysis of variance (ANOVA); r: Pearson correlation coefficient; t: independent samples t-test BMI: body mass index; RLS: restless legs syndrome; SD: standard deviation; TyG: triglyceride-glucose index

Variable	TyG index	Test statistics	p
	Mean ±SD		
Gender		t=-0.153	0.879
Female	8.68 ±0.49		
Male	8.67 ±0.41		
Medication		t=0.496	0.620
Monotherapy	8.69 ±0.47		
Polytherapy	8.65 ±0.49		
RLS severity		F=15.871	0.000* difference: 3>1,2 4>1,2
Grade 1	8.56 ±0.44		
Grade 2	8.48 ±0.45		
Grade 3	8.93±0.41		
Grade 4	8.98±0.38		
		r	p
Age (years)		0.068	0.341
Disease duration (months)		0.281	0.000*
BMI (kg/m^2^)		0.507	0.000*

A multivariate regression analysis was performed to assess the link between the TyG index and the severity, duration, and BMI of RLS in patients (F=26.354, p<0.05). The model is able to account for 33.8% of the observed change in the TyG index (corrected R^2^=0.338). The TyG index was found to be 0.286 times higher in individuals with RLS severity levels 3-4 than in those with RLS severity levels 1-2 (β=0.286, t=4.199, p=0.000). In patients with RLS, there was a statistically significant increase in the TyG index: 0.041 for each unit increase in BMI (β=0.041, t=6.754, p=0.000). In RLS, the TyG index was demonstrated to be a highly significant parameter for increased severity and high BMI (Table 4).

**Table 4:** Multivariate linear regression analysis for the relationship between RLS and TyG *P<0.05; β: regression coefficient; dependent variable: TyG index; F: 26.354, p=0:000*, adjusted R^2^=0,338 BMI: body mass index; CI: confidence interval; RLS: restless legs syndrome; SD: standard deviation; TyG: triglyceride-glucose index

	β (95% CI)	Standard error	t	p
Constant					7.504 (7.205-7.804)	0.152	49.367	0.000
RLS severity (1-2, 3-4)	0.286 (0.152-0.421)	0.068	4.199	0.000*
Disease duration (months)	0.002 (-0.004-0.008)	0.003	0.562	0.575
BMI (kg/m^2^)	0.041 (0.029-0.053)	0.006	6.754	0.000*

**Figure 1:**
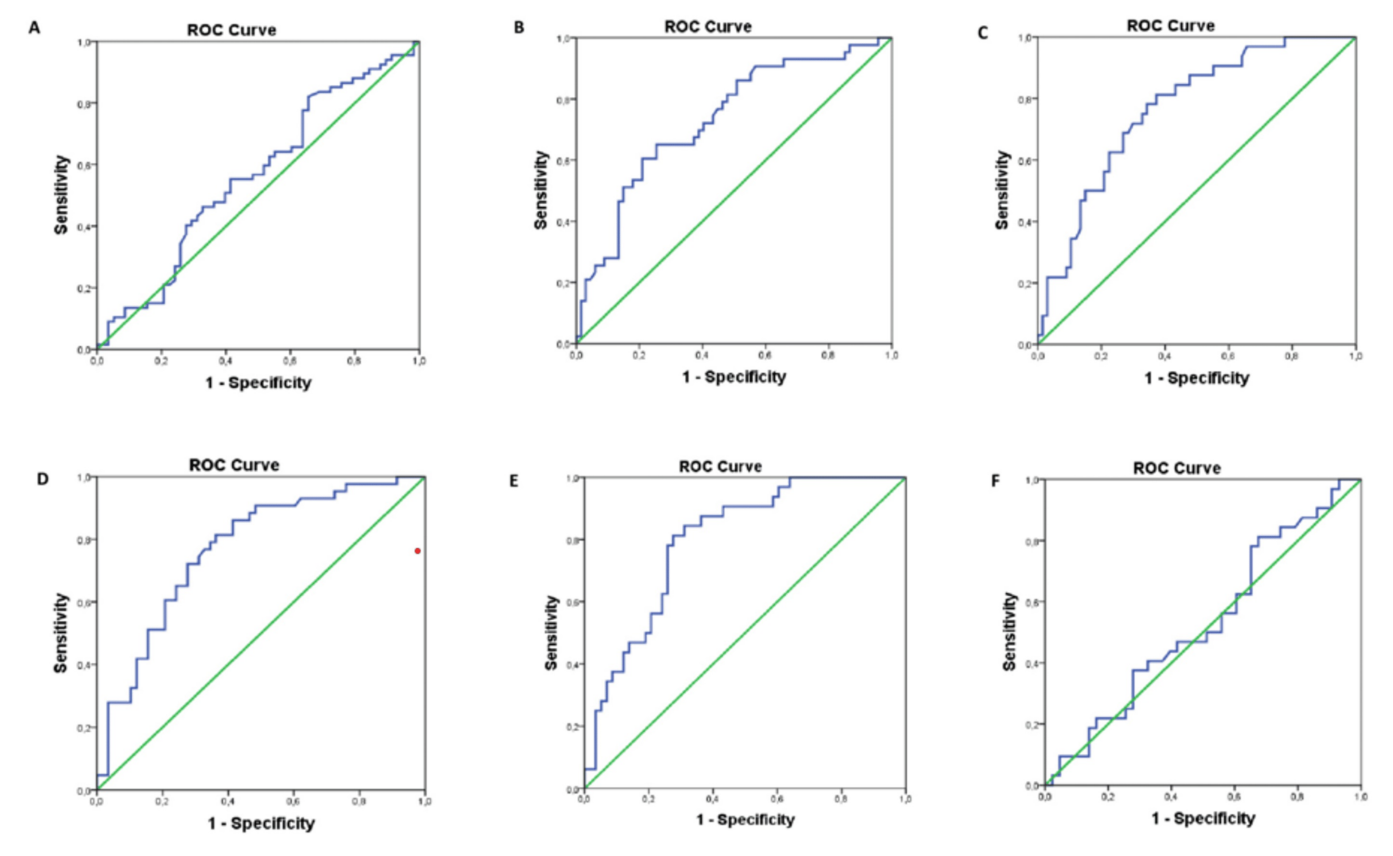
ROC curve of TyG index for RLS severity A: ROC curve of TyG index for RLS severity 1 and 2 (AUC=0.561); B: ROC curve of TyG index for RLS severity 1 and 3 (AUC=0.733); C: ROC curve of TyG index for RLS severity 1 and 4 (AUC=0.766); D: ROC curve of TyG index for RLS severity 2 and 3 (AUC=0.769); E: ROC curve of TyG index for RLS severity 2 and 4 (AUC=0.798); F: ROC curve of TyG index for RLS severity 3 and 4 (AUC=0.528) AUC: area under the receiver operating characteristic curve; RLS: restless legs syndrome; ROC: receiver operating characteristic; TyG: triglyceride-glucose index

The predictive efficiency of the TyG index in RLS severity was evaluated using ROC analysis. The TyG index was a statistically significant discriminator in patients with RLS severity levels 1 and 3 (AUC=0.733, p<0.001) (Figure 1B), in patients with RLS severity levels 1 and 4 (AUC=0.766, p<0.001) (Figure 1C), in patients with RLS severity levels 2 and 3 (AUC=0.769, p<0.001) (Figure 1D), and patients with RLS severity levels 2 and 4 (AUC=0.798, p<0.001) (Figure 1E). However, the TyG index was not found to be a statistically significant discriminator for patients with RLS severity levels 1-2 and 3-4 (AUC=0.561, AUC=0.528, p>0.05) (Figures 1A, 1F).

## Discussion

In the present study, the relationship between idiopathic RLS and the TyG index was evaluated. To the best of our knowledge, this is the first study to investigate the association between the TyG index, severity of the disease, and BMI in RLS. The TyG index in RLS was found to be significantly higher than in healthy controls. In the patient group, a positive correlation was observed between the TyG index and disease severity, disease duration, and BMI. In multivariate regression analyses, it was demonstrated that the TyG index is a remarkably important marker of increased severity and high BMI in RLS.

Although there is increasing evidence that diabetic neuropathy may play a role in the pathophysiology of the increased prevalence of RLS in diabetic patients, the relationship between them is not clearly understood [14]. Researchers have already found that people with RLS are more likely to have neuropathy complications and they are also more likely to have small-fiber neuropathy [7,15]. The fact that small nerve fibers mediate painful neuropathic symptoms suggests that patients with idiopathic RLS may have a prediabetic condition that may cause symptoms.

In our study, we observed that TyG ratios, a simple and reliable index for IR, were significantly elevated in patients with idiopathic RLS. However, we did not observe a difference in FPG values. Similarly, Bosco et al. [16] found that people with RLS were much more likely than controls to have problems with their glucose metabolism after an oral glucose tolerance test (OGTT). Another study on carbohydrate metabolism in sleep disorders demonstrated a notable elevation in mean HbA1c levels and impaired glucose tolerance in patients with RLS [17]. In our study, we analyzed the TyG index according to the disease severity of patients with RLS. Our findings revealed that the TyG index was significantly higher in the severe and very severe (grade 3-4) groups compared to the mild-moderate (grade 1-2) group. Our analyses demonstrated that the TyG index has predictive efficacy in patients with increased RLS severity. Furthermore, we propose that the positive correlation between the TyG index and increased disease duration may be attributed to prolonged exposure to the underlying pathophysiological process.

Obesity is one of the most significant risk factors for developing diabetes. Nevertheless, the evidence indicates that obesity is associated with a reduction in dopamine metabolism in the central nervous system and an increase in vascular pathologies [18,19], indicating that the relationship between obesity and RLS syndrome may be complex and involve multiple mechanisms. Our findings indicated a notable elevation in TG and BMI values in patients diagnosed with idiopathic RLS versus the control group. Similarly, a prospective study with a large sample demonstrated a significant relationship between obesity and high cholesterol levels and a higher risk of developing RLS [20]. Another large cohort study demonstrated that irrespective of the presence of chronic diseases, elevated BMI and waist circumference were associated with an increased prevalence of RLS [21]. In our study, we demonstrated a linear relationship between the increase in BMI and the increase in TyG index in RLS through regression analyses, and we could not identify any similar studies in the literature. It was hypothesized that this relationship was due to obesity increasing the susceptibility to diabetes and impaired glucose metabolism in these patients.

Limitations

This study has a few limitations. Including a larger population could increase the number of RLS patients, but excluding patients with chronic inflammatory and metabolic diseases reduced the number of available patients. Although the patient group was followed up in our outpatient clinic, the fact that the severity of symptoms was self-reported introduces the possibility of minor subjective biases, which would not affect the results.

## Conclusions

Our results indicate a positive correlation between the TyG index and disease severity, disease duration, and BMI in RLS. Our study demonstrated that the TyG index may be a significant marker for the severity of RLS. We recommend further, more comprehensive studies to validate our findings and elucidate the exact mechanisms behind the relationship between TyG and RLS.
